# Supplementary material for: Clustering and reverse transcription of HIV‐1 genomes in nuclear niches of macrophages
Source: EMBO J. 2020 Dec 3;40(1):e105247. doi: 10.15252/embj.2020105247 (PMC7780146; doi:10.15252/embj.2020105247)
Supplement: Supplementary file 2 — Expanded View Figures PDF [file EMBJ-40-e105247-s002.pdf]

Expanded View Figures

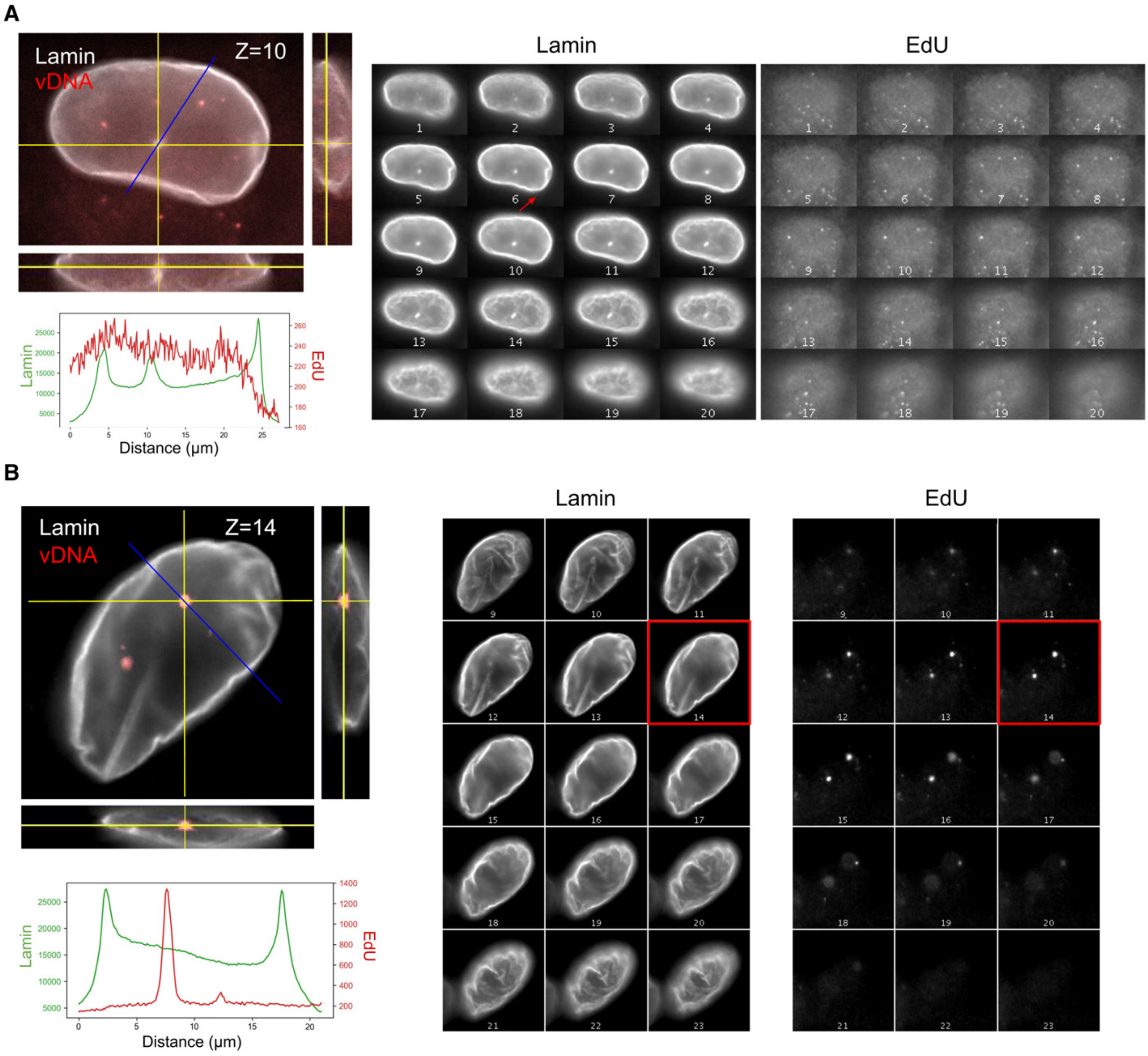

Figure EV1. EdU foci are not located in nuclear invaginations.

A, B 3D images of immunostained lamin and EdU are shown to assess the presence of nuclear envelope invaginations and the location of EdU foci in infected THP-1 cells. Images on the left are orthogonal views of *z*-stacks, where the central image shows an XY slice, and the images to the right and below show XZ and YZ slices at positions indicated by the yellow lines. The blue line indicates the location for the measurement of intensity profiles. The plots below show intensity profiles for the lamin and EdU channel along the blue lines. Montage views on the right show all *z*-slices of the Lamin and EdU channels separately. (A) Nucleus of an infected cell showing an invagination, as evidenced by the enrichment of Lamin in the intensity profile (middle peak), as well as in the montage views. The chosen line profile crosses the invagination but does not show an enrichment of EdU. (B) Another infected cell with a line profile chosen to cross one of the EdU foci. No lamin enrichment was detected at the location of the EdU foci (peak of EdU). Panels on the left are identical to Fig 1C.

© 2020 The Authors

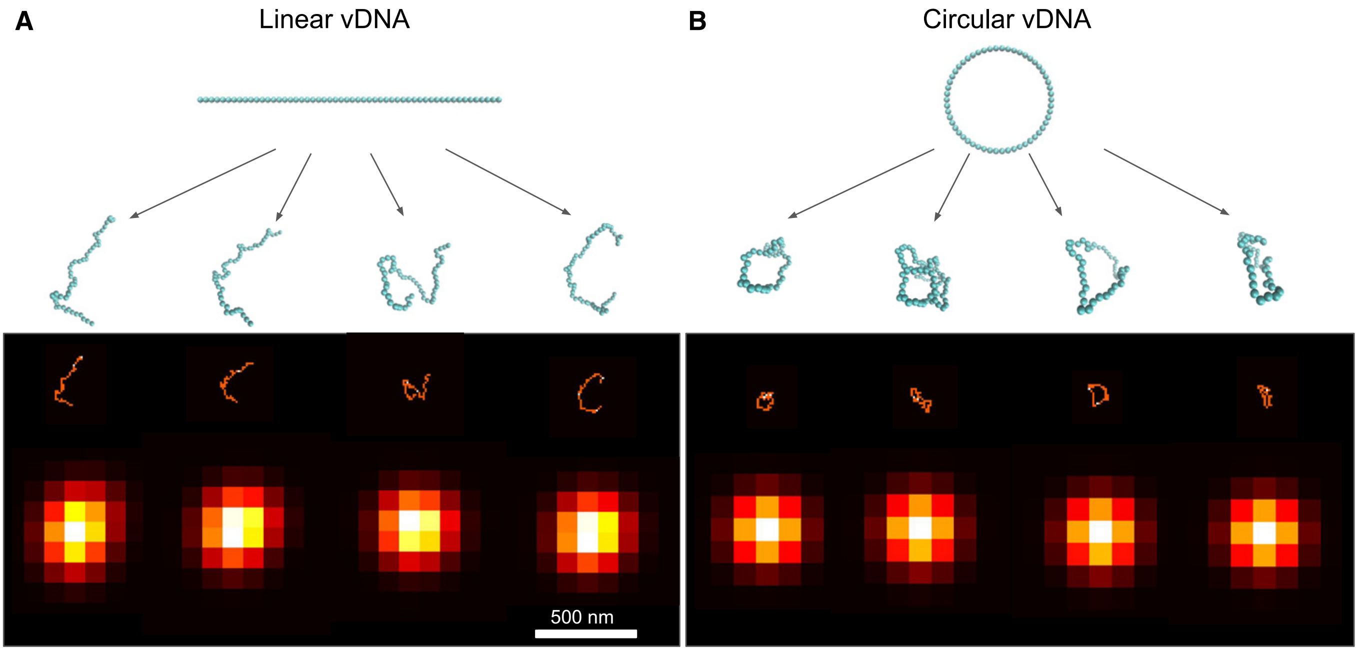

Figure EV2. Simulated configurations and images of HIV-1 DNA.

A, B Simulated configurations and images of the HIV-1 DNA in linear (A) or circular form (B). The 10 Kb long viral genome is represented as a chain of 59 nucleosomes of 11 nm diameter. Starting from the linear or circular initial structures shown (blue chains on top), molecular dynamics simulations (Langevin dynamics) generate 100 independent configurations (only 4 are shown as blue chains of beads). The corresponding image is blurred by convolution with the microscope point spread function (approximated as a Gaussian of standard deviation 100 nm), resulting in the images shown in the bottom row.

 © 2020 The Authors

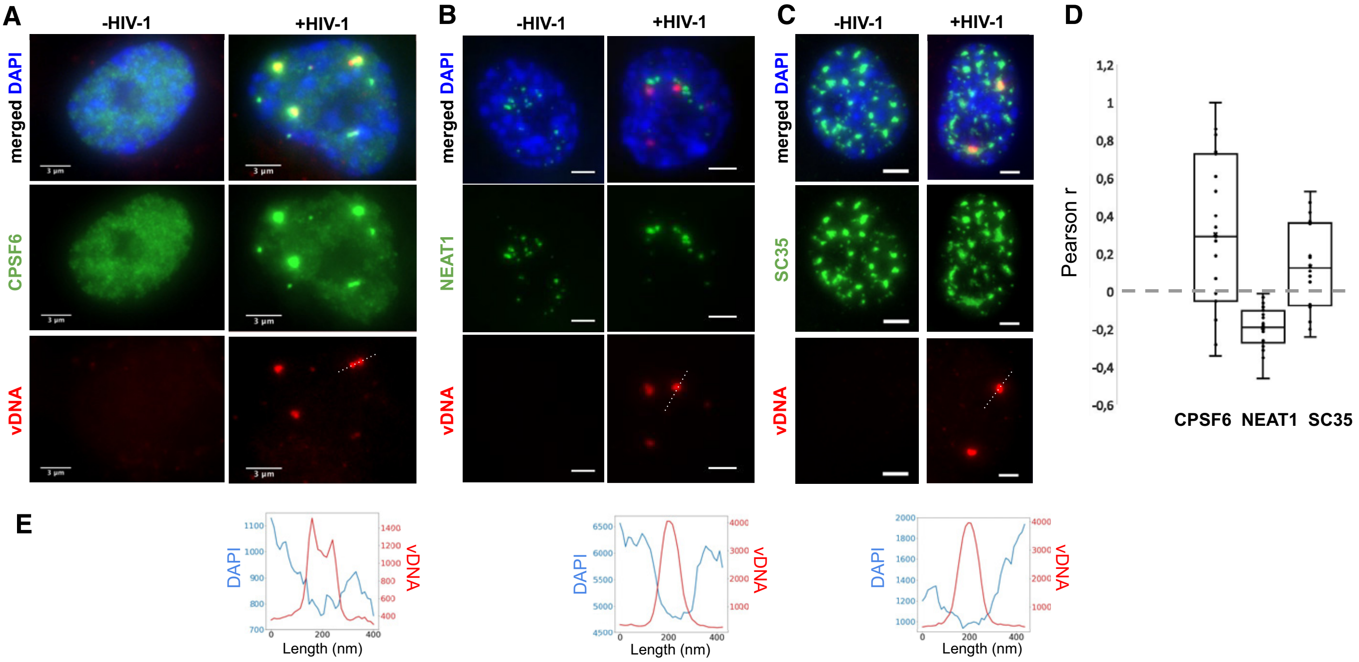

Figure EV3. vDNA clusters do not colocalize with the CA-binding protein NONO.

A, B Image of uninfected (left) and infected (right) THP-1 cells showing immunolabeled NONO in green, EdU in red, and the nucleus (DAPI) in blue. Scale bars: 15 µm in (A), 5 µm in (B).

C Intensity profiles of EdU, DAPI, and NONO along a profile crossing a vDNA focus (rectangle in B).

D Boxplot shows Pearson correlations between vDNA (EdU) and NONO. Center line defines the median, top and bottom limits define upper and lower quartiles, respectively. Whiskers show full data range, except for outliers. Black dots are individual data points. The negative correlation values indicate an absence of colocalization between vDNA and NONO (Costes *P*-value < 0.05 for 18 out of 18 cells, and < 0.01 for 14 out of 18 cells; data for one experiment).

© 2020 The Authors

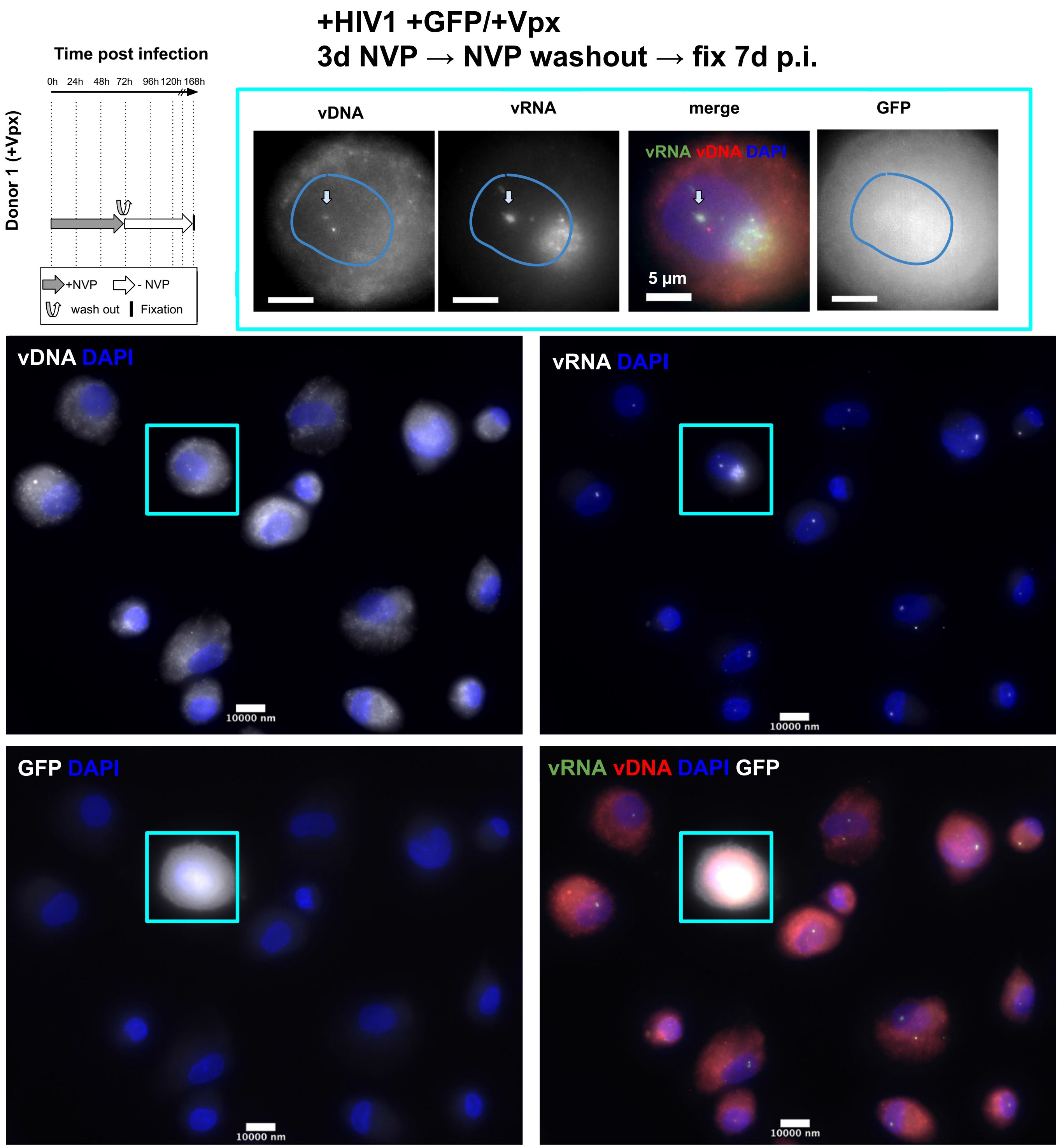

Figure EV4. HIV-1 transcription in primary macrophages after temporary inhibition of RT.

Multicolor images of MDMs from donor 1, infected with a HIV-1 virus carrying a GFP reporter and in presence of Vpx. Cells were treated with Nevirapine (NVP) for 3 days, then NVP was washed out and cells were fixed for imaging 4d later (i.e., at 7 days p.i.). Images show the vDNA, vRNA and/or GFP signal and DAPI.

© 2020 The Authors
